# Phenylboronic acid-functionalized magnetic nanoparticles for one-step saccharides enrichment and mass spectrometry analysis

**DOI:** 10.1007/s41048-015-0002-3

**Published:** 2015-07-14

**Authors:** Xiangdong Xue, Yuanyuan Zhao, Xu Zhang, Chunqiu Zhang, Anil Kumar, Xiaoning Zhang, Guozhang Zou, Paul C. Wang, Jinchao Zhang, Xing-Jie Liang

**Affiliations:** CAS Key Laboratory for Biomedical Effects of Nanomaterials and Nanosafety, National Center for Nanoscience and Technology, Beijing, 100190 China; Laboratory of Pharmaceutics, School of Medicine, Tsinghua University, Beijing, 100084 China; College of Chemistry & Environmental Science, Chemical Biology Key Laboratory of Hebei Province, Hebei University, Baoding, 071002 China; Fu Jen Catholic University, Taipei, 24205 China

**Keywords:** Magnetic nanoparticles, MALDI-TOF MS, Glucose, *N*-Glycans, Oligosaccharides

## Abstract

**Abstract:**

In this work, 2-(2-aminoethoxy) ethanol-blocked phenylboronic acid-functionalized magnetic nanoparticles (blocked PMNPs) were fabricated for selective enrichment of different types of saccharides. The phenylboronic acid was designed for capturing the *cis*-diols moieties on saccharides molecules, and the 2-(2-aminoethoxy) ethanol can deplete the nonspecific absorption of peptides and proteins which always coexisted with saccharides. For mass spectrometry analysis, the PMNPs bound saccharides can be directly applied onto the MALDI plate with matrix without removing the PMNPs. By PMNPs, the simple saccharide (glucose) could be detected in pmol level. The complex saccharides can also be reliably purified and analyzed; 16 different *N*-glycans were successfully identified from ovalbumin, and the high-abundance *N*-glycans can be detected even when the ovalbumin was in very low concentration (2 μg). In human milk, ten different oligosaccharides were identified, and the lactose can still be detected when the human milk concentration was low to 0.01 μL.

**Graphical Abstract:**

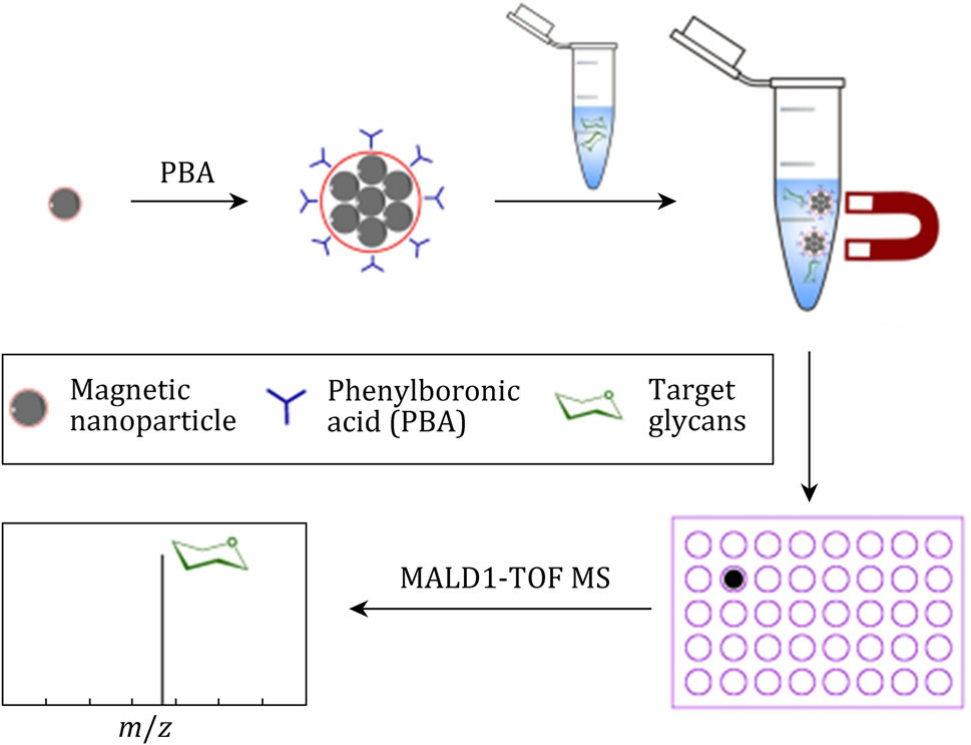

**Electronic supplementary material:**

The online version of this article (doi:10.1007/s41048-015-0002-3) contains supplementary material, which is available to authorized users.

## **INTRODUCTION**

Glycosylation is one of the most common and important biological phenomena of post-translational modification processes in eukaryotic cells, playing a vital role in the modulation of protein functions (Wang et al. 2006). A growing body of evidence indicates that alterations of protein glycosylation (Ohtsubo and Marth 2006) are associated with various diseases and abnormal physiological conditions, including inflammation, (Arnold et al. 2008) immune diseases, (Marth and Grewal 2008) tumors, (Goetz et al. 2009) and metabolic syndromes (Sun et al. 2005). Moreover, glucose and oligosaccharides are important energy sources for human and animals. Therefore, analysis of those saccharides from biological samples is important, yet full of challenges since it involves a series of tedious and complex biochemical operations. The traditional saccharides separation method is affinity chromatography, (Shimizu et al. 2001) which is not only time consuming and sophisticated but also susceptible to lose targeted samples, for this reason, the affinity chromatography is not suitable for analysis of low-abundant saccharides samples.

With the development of nanotechnology, different kinds of nanoparticles have been employed for the detection of biological samples (Nash et al. 2012; Wang et al. 2012; Zhang et al. 2012). Magnetic nanoparticles (MNPs) provide a highly efficient and easily operative method in the separation of biological samples, controlled by an assistant magnetic field (Bao et al. 2007). Magnetic nanoparticles separation has been implemented in many applications: Joshua E. Smith and his coworkers (Medley et al. 2011) conjugated aptamer onto the surface of MNPs to detect cancer cells; and Chun-Cheng (Lin et al. 2006) modified specific antibody onto the MNPs, and successfully enriched the targeted proteins from plasma. Phenylboronic acid-functionalized nanoparticles have been extensively used for glucose detection (Kaur et al. 2006; Fang et al. 2004) and glycopeptides enrichments (Xu et al. 2008; Lin et al. 2011; Zhang et al 2009, 2012b; Zhou. et al. 2008). However, fewer works about pure saccharide-specific magnetic enrichment and analysis have been reported, because saccharides have lower ionization efficiency in mass spectrometry analysis, as compared to their high-abundant interferents, like peptides and proteins. And these interferents always coexist with saccharides; it is detrimental to pure saccharides detection. To analyze saccharides by mass spectrometry, the peptides and proteins must be removed exhaustively, as their ion peaks will strongly depress the peaks of saccharides.

Herein, we describe a convenient and highly efficient magnetic solution that can selectively enrich saccharides in one step for glycomic analysis. In this approach, 2-(2-aminoethoxy) ethanol-blocked phenylboronic acid-functionalized Fe_3_O_4_ magnetic nanoparticles (blocked PMNPs) with high magnetic responsibility were fabricated. The phenylboronic acid (PBA) group can specifically recognize the *cis*-diols moieties of saccharides, forming a reversible covalent boronate ester bond (Kaur et al. 2006; Springsteen and Wang 2002; Yan et al. 2004). The blocking molecule, 2-(2-aminoethoxy) ethanol, can deplete nonspecific binding during the separation process. The enriched saccharides were detected by matrix-assisted laser desorption ionization time-of-flight mass spectrometry (MALDI-TOF MS), which is a high-throughput platform with high sensitivity, (Caprioli et al. 1997; Franc et al. 2012) desired salt tolerance, and allowing for detailed analysis of saccharides structures (Jeong et al. 2012; Selman et al. 2012). Unlike currently used methods, the enriched saccharides on the PMNPs can be directly analyzed by MALDI-TOF MS without extra steps to remove the PMNPs. For saccharides analysis, the PMNPs method was utilized to isolate different types of saccharides, including simple sugar, *N*-glycans, and oligosaccharides.

## **EXPERIMENTAL SECTION**

### **Materials and reagents**

FeCl_2_·4H_2_O, FeCl_3_·6H_2_O, malonic acid, 2-(7-Aza-1H-benzotriazole-1-yl)-1,1,3,3-tetramethyluronium hexafluorophosphate (HATU), *N*,*N*-diisopropylethylamine (DIEA), and dimethylformamide (DMF) were from Bomaijie Inc. (Beijing, China). Glucose, glutamine, ammonium hydroxide (28~30 wt%), tetraethylorthosilicate (TEOS), (3-aminopropyl) triethoxysilane (APS), 2-(2-aminoethoxy) ethanol, 4-(*trans*-2-carboxyvinyl) phenylboronic acid, and ovalbumin were purchased from Sigma-Aldrich (St. Louis, MO, USA). Water was purified on a Milli-Q system (Millipore, Milford, MA, USA). PNGase F was purchased from New England Lab. Human milk samples were provided by a healthy mother, attending the First Affiliated Hospital of Medical College, Peking University, China.

### **Instrument**

MALDI-TOF MS (Microflex LRF, Bruker Daltonics), X-ray diffractometer (D/MAX-TTRIII, Rigaku Corp.), Physical performance analyzer (PPMS-9, Quantum Design Inc.), FTIR spectroscopy (Spectrum one, Perkin Elmer), Zetasizer (Nano ZS90, Malvern), and transmission electron microscope (TEM, Tecnai G2 20 S-TWIN) were used.

### **Fabrication of 2-(2-aminoethoxy) ethanol-blocked PBA-functionalized Fe3O4 magnetic nanoparticles (PMNPs)**

**Scheme 1:**
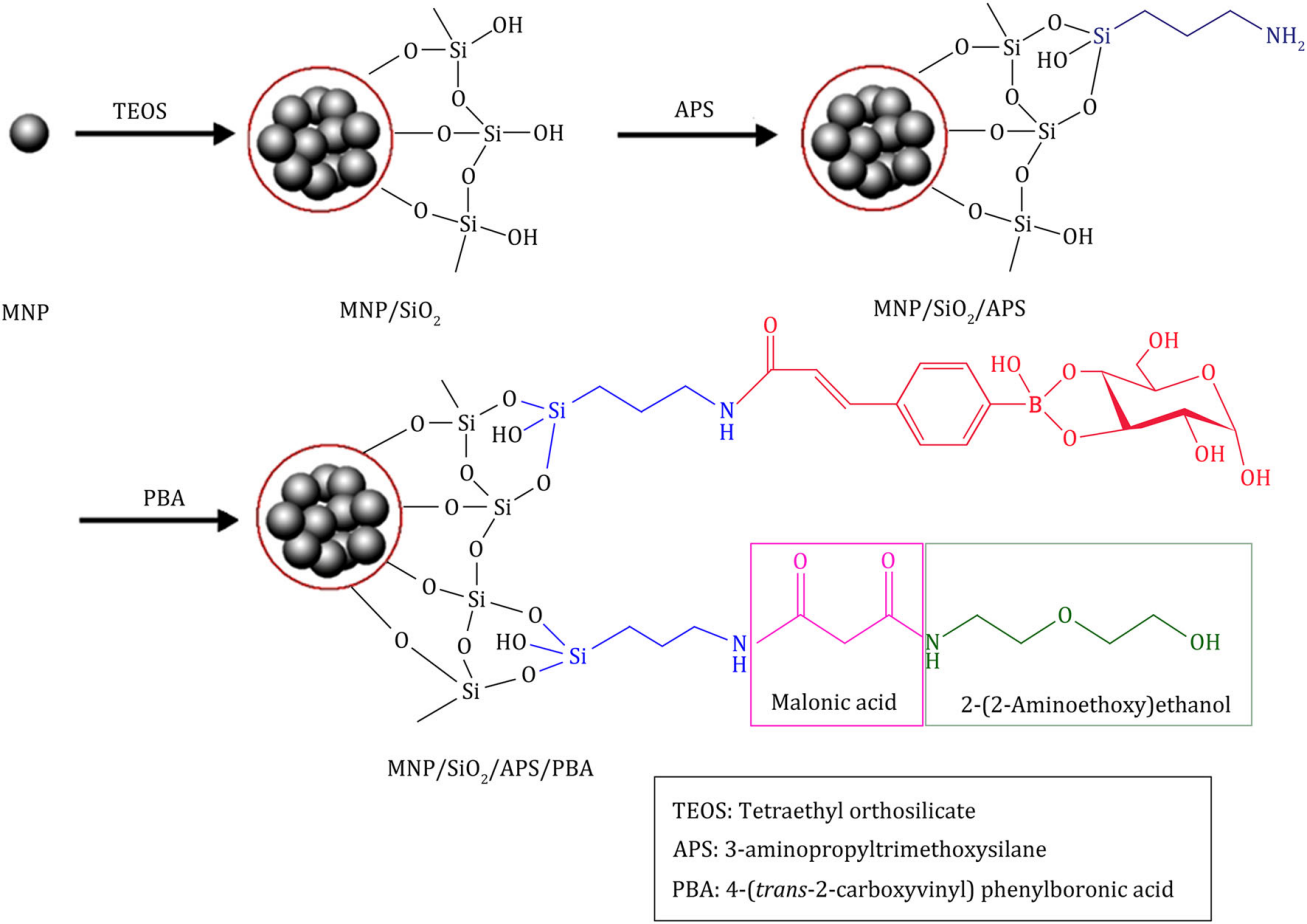
Schematic representation of fabrication of the 2-(2-aminoethoxy) ethanol-blocked PBA-functionalized magnetic nanoparticles

The fabrication processes are illustrated in Scheme 1. Firstly, the Fe_3_O_4_ magnetic nanoparticles (MNPs) were prepared by typical coprecipitation method. (Chen and Chen 2005; Kim et al. 2003) 2 g FeCl_2_·4H_2_O (10 mmol) and 5.4 g FeCl_3_·6H_2_O (20 mmol) were dissolved in 25 mL HCl (2 mol/L), and 30 mL ammonia solution was then added to the mixture dropwise and stirred vigorously for 30 min at ambient temperature. The whole procedures were under N_2_ protection by continuously passing nitrogen through the reaction system. The Fe_3_O_4_ MNPs were rinsed with Milli-Q water (50 mL) three times and suspended in 50 mL Milli-Q water. The concentration of the Fe_3_O_4_ MNPs was set at 40 mg/mL.

The Fe_3_O_4_ MNPs suspension (5 mL, 40 mg/mL) was separated by a constant magnetic force, dispersed in 80 mL ethanol and then sonicated for 10 min. 8 mL ammonia solution, 7.5 mL water, and 0.1 mL TEOS were added to the MNPs solution sequentially, and stirred vigorously at 40 °C for 4 h; SiO_2_ was doped into Fe_3_O_4_ MNPs. The SiO_2_ doped MNPs were then rinsed with 50 mL water three times and dissolved in 60 mL ethanol.

20 mL of SiO_2_-doped Fe_3_O_4_ MNPs suspension was sonicated for 30 min, and then 1 mL of ammonia solution and 0.03 mL of APS were added to the solution dropwise and stirred vigorously under ambient temperature for 24 h; amino groups were modified onto the surface of MNPs. The aminated surface-modified MNPs were rinsed with 30 mL DMF for three times, and dispersed in 30 mL DMF for next modification.

Further, 3 mL aminated MNPs were sonicated for 30 min, and then 5 mL HATU solution (0.5 mol/L in DMF), 2.5 mmol 4-(*trans*-2-carboxyvinyl) phenylboronic acid, and 5 mmol DIEA were added to the reaction system sequentially. After vigorous stirring for 0.5 h, 2.5 mmol malonic acid was added to the solution, and then the reaction system was vigorously stirred for 24 h in 4 °C. In order to block the nonspecific binding of peptides to the PMNP, 2.5 mmol 2-(2-aminoethoxy) ethanol was added to the mixture and followed by shaking for 24 h at 4 °C. For fabrication of the non-blocked PMNPs, steps of adding malonic acid were avoided, and instead, 5 mmol 4-(*trans*-2-carboxyvinyl) phenylboronic acid with 5 mL HATU solution (0.5 mol/L in DMF) and 5 mmol DIEA was directly added to the aminated MNPs suspensions, and the reaction system was shaken overnight at 4 °C. With magnetic separation, the PBA-functionalized magnetic nanoparticles (PMNPs) were washed with 5 mL Milli-Q water 5 times and stored at 4 °C. The concentration of PMNPs was set at 1.0 mg/mL.

### **N-glycans enzymolysis from ovalbumin**

The *N*-glycans enzymolysis step was carried out by dissolving 1 mg of ovalbumin in 200 μL glycoprotein denaturing buffer, and denatured at 100 °C for 10 min. After cooling down the denaturing buffer to room temperature, 20 μL 10 % NP-40, 20 μL 10 % G7 reaction buffer, and 2 μL PNGase F (500,000 units/mL) were added sequentially, then reaction mixture was incubated for 24 h at 37 °C. After that the product was lyophilized and used for further experiment.

### **Human milk pretreatment**

The human milk samples were taken 30 days after parturition, and stored at −20 °C until to be used. The whole milk was completely thawed prior to centrifugation at 13,000 rpm for 30 min at 4 °C, and the upper layer and precipitation was discarded, which contained fats and proteins.

### **Mass spectrometry analysis**

All mass spectra were collected by MALDI-TOF mass spectrometer (Microflex LRF, Bruker Daltonics) equipped with a 337 nm nitrogen laser source. Measurements were taken in reflect, positive ion mode at 25 kV acceleration voltage, first grid at 70 % of acceleration voltage, pulse ion extraction at 100 ns. 500 single shots spectra were accumulated to improve the signal-to-noise ratio. 2,5-dihydroxybenzoic acid (DHB) was employed as matrix according to the manufacture’s recommendation.

### **Schematic specific enrichment of saccharides by PMNPs and analysis by MALDI-TOF MS**

The analytical process of our one-step saccharides enrichment and analysis is demonstrated in Figure S1. In brief, PBA was modified onto the surface of the MNPs to form a special *cis*-diols affinity beads—PBA-functionalized MNPs (PMNPs). For the enrichment of saccharides, PMNPs were drawn from the 100 μL suspension (1 mg/mL) with an assistant magnetic field, and then resuspended in alkalescent buffer (ammonia solution, pH 8.5). The PMNPs in alkalescent suspension were sonicated for 10 min to make them dispersed sufficiently, incubated with the biological samples for 10 min under ambient temperature, and then rinsed the PMNPs with alkaline buffer 3~4 times. After being rinsed, the PMNPs was dispersed in Milli-Q water, spotted onto the MALDI-TOF MS plate with matrix, and analyzed by mass spectrometry.

## **RESULTS AND DISCUSSION**

### **Characterization of phenylboronic acid-functionalized magnetic nanoparticles**

**Fig. 1:**
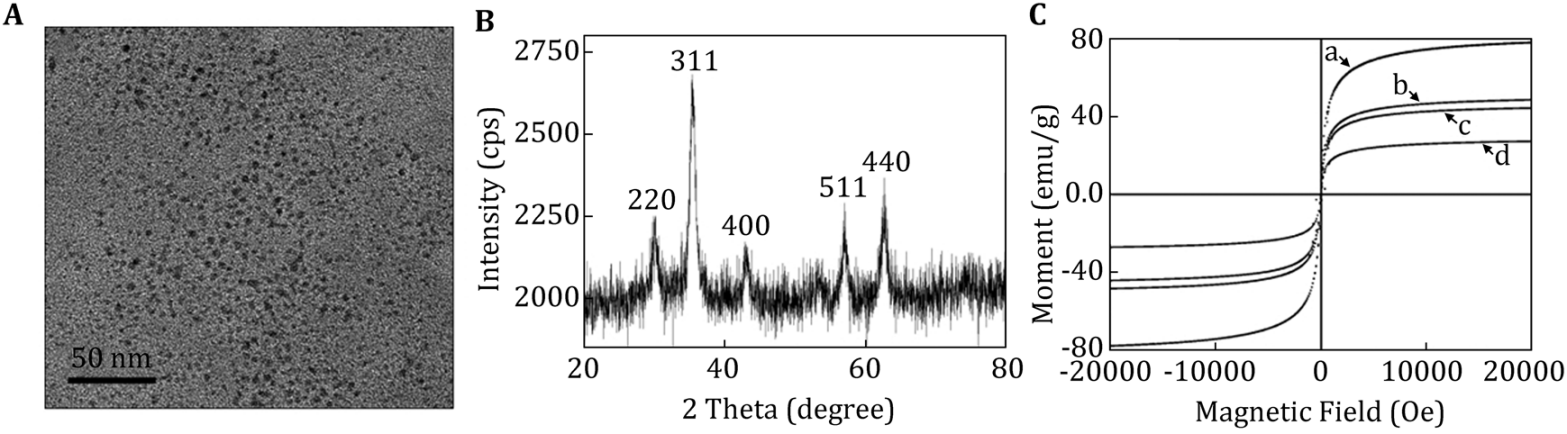
Characterization of Fe_3_O_4_ MNPs. **A** TEM image of Fe_3_O_4_ MNPs, **B** X-ray diffraction pattern of Fe_3_O_4_ MNPs, **C** magnetization curve of MNPs measured by M-H hysteresis loop behavior at 300 K: (1) Fe_3_O_4_ MNPs, (2) Fe_3_O_4_/SiO_2,_ (3) Fe_3_O_4_/SiO_2_/APS, (4) the blocked PMNPs

The morphology of Fe_3_O_4_ nanoparticles was examined by TEM, the TEM image (Fig. 1A) showed that the size of the Fe_3_O_4_ nanoparticles was around 8 nm. As shown in Fig. 1B, there were five different characteristic peaks (exact peak position), corresponding to the indices (2 2 0), (3 1 1), (4 0 0), (5 1 1), and (4 4 0) of Fe_3_O_4_ nanoparticles (Table S1, PDF card no. 19-0629). The X-ray diffraction result indicated that the obtained Fe_3_O_4_ nanoparticles were with a face-centered anti-spinel structure.

The M-H hysteresis loop behavior of Fe_3_O_4_ MNPs was measured at 300 K by cycling the field between −20,000 and 20,000 Oe. As shown in Fig. 1C (1), there was no remanence or coercivity in the hysteresis, which indicated the typical superparamagnetic characteristics of Fe_3_O_4_ MNPs. Also, the saturation magnetization was 75.8 emu/g.

**Fig. 2:**
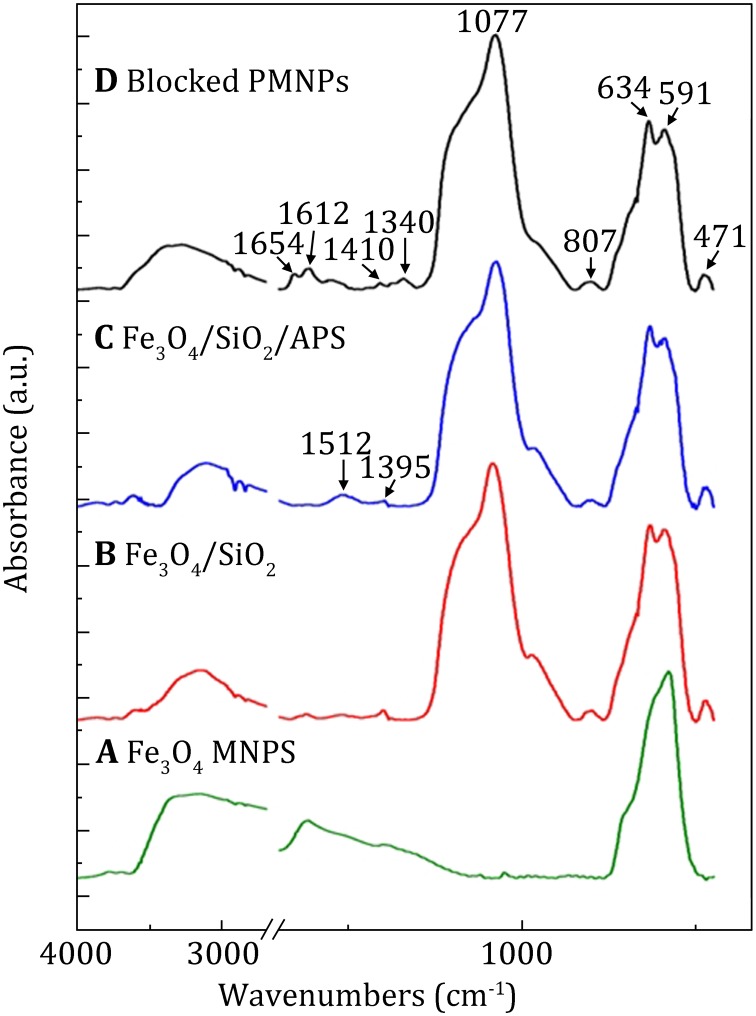
FTIR spectra of* A* Fe_3_O_4_ MNPs,* B* Fe_3_O_4_/SiO_2,_ *C* Fe_3_O_4_/SiO_2_/APS,* D* blocked PMNPs

The processes of MNPs modification were evaluated by FTIR, and the spectra of Fe_3_O_4_ MNPs, Fe_3_O_4_/SiO_2_, Fe_3_O_4_/SiO_2_/APS and the blocked PMNPs are shown in Fig. 2A–D. The absorption band at 591 cm^−1^ corresponded to the metal–oxygen band in tetrahedral and octahedral sites (Fig. 2A). The bands existed at 807 and 1077 cm^−1^ were assigned to Si–O–Si skeleton vibrations (Fig. 2B). Besides Si–O–Si skeleton vibration, an additional absorption peak at 1512 cm^−1^, corresponding to *δ* (NH), was observed, (Zhao et al. 2010) suggesting the successful modification of APS onto Fe_3_O_4_/SiO_2_ (Fig. 2C). In Fig. 2D, the absorption peaks at 1654 and 1551 cm^−1^ were ascribed to the characteristic vibrations of amide I and amide II, respectively; (Hamm et al. 1998; Dousseau and Pezolet 1990) the bond at 1612 cm^−1^ was assigned to *v* (C=C) of aromatic ring; (Hu et al. 2002) and these bands indicated that PBA and blocking molecule were linked onto the surface of Fe_3_O_4_/SiO_2_/APS. Also, the absorption peak at 1340 cm^−1^ was ascribed to *v*_s_ (B–O), (Tiwari et al. 2010) which further confirmed that the boronic acid moieties had been introduced successfully.

**Fig. 3:**
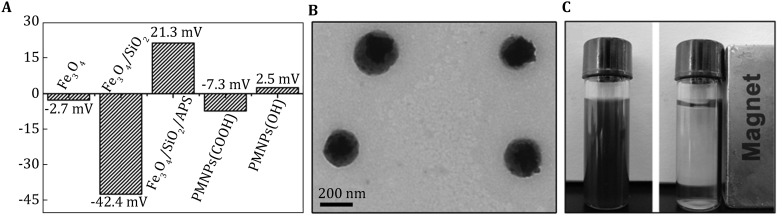
**A** ζ-potentials of Fe_3_O_4_ MNPs, Fe_3_O_4_/SiO_2_, Fe_3_O_4_/SiO_2_/APS, PMNPs (COOH), and PMNPs (OH), **B** TEM image of PMNPs, **C** photograph showing the separation of PMNPs from the solvent

ζ-potential was employed to demonstrate the surface charge changes with the modifications of MNPs. As shown in Fig. 3A, after being doped into SiO_2_, the ζ-potential value of Fe_3_O_4_ MNPs (−2.7 mV) changed to −42.4 mV, which was the same to pure silica nanoparticle, indicated the successful doping of magnetic nanoparticles into SiO_2_. After being modified by APS, the ζ-potential value became positive (21.3 mV), due to the protonation of amino groups on the surface of the Fe_3_O_4_/SiO_2_/APS. When malonic acid and PBA were introduced, the positively charged Fe_3_O_4_/SiO_2_/APS turned into a negative value (−7.3 mV), indicating that the surface of MNPs was partly carboxylated. Finally, ζ-potential value changed to 2.5 mV after modification of blocking molecule. Combining with the ζ-potential and IR results, the blocked PMNPs were successfully fabricated. All ζ-potential experiments were conducted at neutral pH and the solvent was Milli-Q water.

TEM image of PMNPs is demonstrated in Fig. 3B. There are dozens of darker spots corresponding to the Fe_3_O_4_ nanoparticle in every single particle, which implies the strong magnetic field response of obtained nanomaterials. Vibrating sample magnetometer (VSM) spectra showed that the saturation magnetization of the nanoparticles decreased accordingly; when it had been doped into silica nanoparticles and experienced further surface modification by APS and PBA, the resulted PMNPs demonstrated superparamagnetic property (Fig. 1C (2–4)); after the modification, the saturation magnetization of the blocked PMNPs was 26.6 emu/g. And it could be easily dispersed in alkaline buffer and completely drawn from the solution to the sidewall of the vial by a magnet field within around 10 s (Fig. 3C). The obtained superparamagnetic PMNPs would be used directly for the detection of saccharides molecules.

Based on the characterization results, the blocked phenylboronic acid-functionalized MNPs were successfully fabricated. The phenylboronic acid can specifically recognize and form a reversible boronate ester with *cis*-diols moieties, including on a sugar ring (Dowlut and Hall 2006) (Figure S2). The formation of reversible boronate ester is reversible and pH dependent. Boronic acid and saccharides can form reversible boronate ester under basic circumstance, and be hydrolyzed into boronic acid and saccharide in neutral or weak acid solution. Therefore, the selective saccharides recognition ability of the boronic acid moieties on the surface of the MNPs would be studied by the enrichment of the simple sugar molecule and complex saccharides.

### **Validation the PMNPs on isolating glucose**

**Fig. 4:**
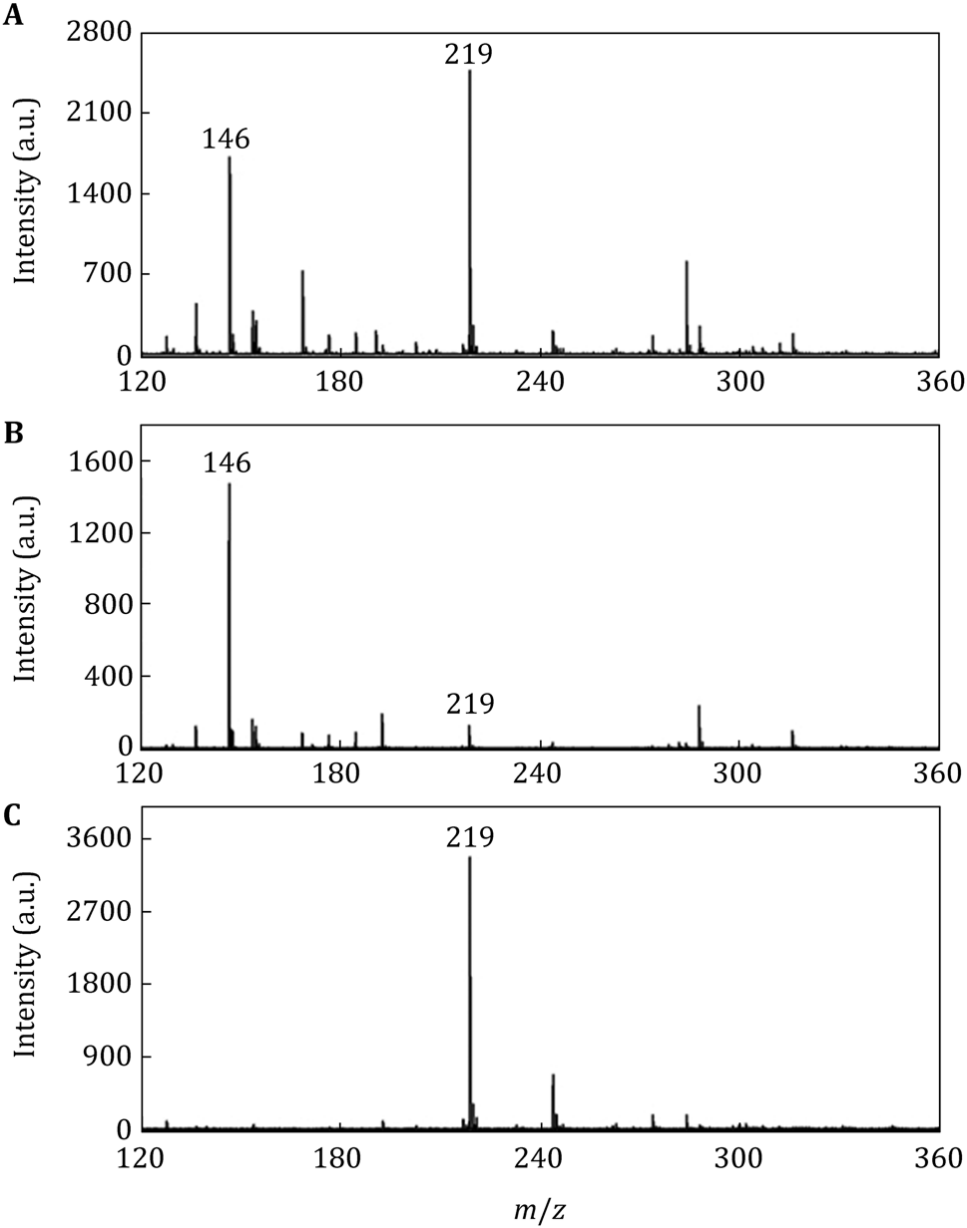
Separation of glucose from glutamine by the blocked PMNPs and detected by MALDI-TOF MS: **A** mixture of glucose and glutamine at the molar ratio of 1:1 (10^−6^ mol), **B** supernatant of the mixture after being treated with PMNPs, **C** glucose associated with PMNPs

For simple sugar enrichment and analysis, glucose was chosen as standard to evaluate the selectivity of PMNPs. To mimic the selective enrichment of the glucose from other interferents, we designed to premix the same amount (10^−6^ mol) of glucose and glutamine together for PMNP enrichment and analysis, the results are shown in Fig. 4. Before separation (Fig. 4A), both glucose and glutamine can be detected: *m/z* 146 was the [M+H]^+^ ion peak of glutamine, and *m/z* 219 was the [M+K]^+^ ion peak of glucose. After being separated by the blocked PMNPs (100 μg), as shown in Fig. 4B, the major species in the elution was glutamine, only a small amount of glucose was detected. The rinsed PMNPs were spotted on to the MALDI-TOF plate, as shown in Fig. 4C, only glucose ion peak was detected, indicating that the blocked PMNPs could effectively extract glucose from glutamine.

To investigate the influence of “blocking” molecules for isolating simple sugar sample, the non-blocked PMNPs were applied to isolate glucose from glutamine. As shown in Figure S3, the glucose also can be isolated. It seemed that the “blocking” molecules showed no influence to the isolation of glucose. Because in the simple samples, which contained less interferents, the nonspecific absorptions of peptides or proteins are not existed, the glucose can be isolated by the non-blocked PMNPs. The non-blocked PMNPs would be applied to isolate complex glycans from biological samples to further study whether the “blocking” molecules can deplete the nonspecific absorption of peptide or proteins.

Furthermore, 100 μg blocked PMNPs were evaluated by selectively separating glucose from the mixtures (1:1, mol/mol) of glucose and glutamine in different concentrations. As shown in Figure S4, when the concentration of the mixture reached to 10^−10^ mol, the ion peak of glucose could be detected (Figure S4 A). Then, the concentration of mixture was decreased to pmol (10^−12^ mol), the ion peak of glucose still could be observed, but the intensity was lower as compare to ion peak of matrix, which suppressed by the ion peak from matrix (Figure S4 B). When the concentration of mixture was reduced further, less than pmol level (10^−14^ mol), the glucose ion peak could not be identified, almost covered by the peak of matrix (data not shown). The high sensitivity of the glucose isolation may be caused by two reasons: (i) Boronic acid moieties on the surface of PMNPs can bind to the equal molar of *cis*-diols. Thus, saccharides with multi-*cis*-diols will bind to multiple PBA molecules. For the simple sugar molecules, such as glucose, which has two *cis*-diols on the sugar ring, making them easier to bind to the boronic acid moieties, and thus lower the detection limits of the PMNPs method; (ii) The MALDI-TOF MS also an important technique to improve the detection sensitivity.

### **Validation the PMNPs on*N*-glycans from ovalbumin**

**Fig. 5:**
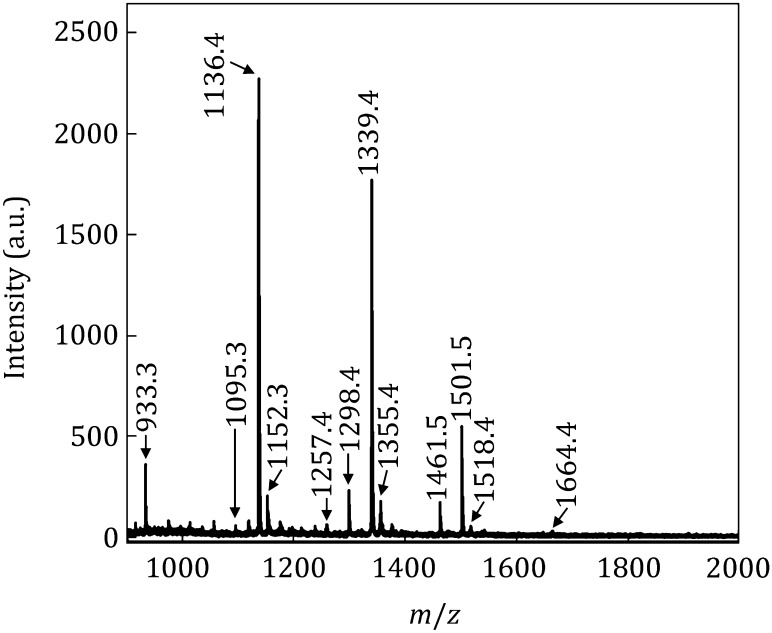
MALDI mass spectrum of *N*-glycans from 10 μg ovalbumin purified by 100 μg blocked PMNPs

For complex saccharides samples enrichment and analysis, *N*-glycans from ovalbumin were chosen as standard to evaluate the selectivity of PMNPs. The blocked PMNPs (100 μg) were employed to analyze *N*-glycans from 10 μg ovalbumin. The blocked PMNPs can enrich glycans efficiently, ion peaks of the isolated *N*-glycans were clearly shown in MS spectrum (Fig. 5), 13 main ion peaks of glycans were observed, corresponding to 11 *N*-glycans.

To investigate the influence of “blocking” molecules for isolating complex glycans molecules, the non-blocked PMNPs were applied to isolate *N*-glycans from ovalbumin. As shown in Figure S5, the influence of the “blocking” molecules was obviously. The non-blocked PMNPs were not able to enrich the *N*-glycans efficiently, just a few glycans ion peaks were observed, and they were strongly depressed by the ion peaks of the nonspecific absorbed peptides. The MS results (Fig. 5; Figure S5) indicated that the “blocking” molecules can deplete the nonspecific absorptions effectively. The influence of the “blocking” molecules indicated that the binding of saccharides and PMNPs are associated with the compositions of the samples and nano-environment of PBA. For glucose isolation, because it is standard samples without other absorbed competitors, the glucose can be isolated by the non-blocked PMNPs; but for the biological samples, the glycans always coexisted with the peptides or proteins, PMNPs without “blocking” molecules could absorb these interferents, the ion peaks of the peptides would strongly depress the peaks of glycans. The PMNPs with “blocking” molecules on their surface could isolate the glycans from biological samples efficiently, because the “blocking” molecules can deplete the nonspecific absorptions of peptides.

Furthermore, different contents of ovalbumin were enriched by 100 μg blocked PMNPs. 40 μg of PNGase F digested ovalbumin was enriched and analyzed by PMNPs method. As shown in MS spectrum (Figure S6), more ion peaks of *N*-glycans were observed (*m/z* 1419.1, *m/z* 1705.5, *m/z* 1745.8, *m/z* 1866.8, *m/z* 1908.2, and *m/z* 1949.2). But the ion peaks were not as clear as that isolated by 100 μg blocked PMNPs; the reason may be that the increase of glycoproteins would improve the probability of absorbing the nonspecific peptides. Then, 2 μg ovalbumin was enriched by 100 μg PMNPs, as shown in Figure S7, just three glycans in high abundance were observed (*m/z* 1136.4, *m/z* 1339.4 and *m/z* 1501.5), since the concentration of ovalbumin was too low. For complex *N*-glycan enrichment and analysis, because of their steric hindrance and rigidity of the sugar molecular chains, boronic acid moieties may not bind to *cis*-diols quantitatively, the PMNPs method was not as efficient as the analysis of glucose.

In PMNPs enrichment and analysis experiments, 18 ion peaks of glycans from ovalbumin were observed, corresponding to 16 *N*-glycans. The compositions of the enriched *N*-glycans are concluded in Table 1. All the glycans had a core “3[Hex]2[HexNAc]”, which was the common structure of *N*-glycans.

**Table 1:** *N*-glycans compositions of ovalbumin isolated by the blocked PMNPs

*m/z* [M+Na]^+^	Glycans compositions	*m/z* [M+Na]^+^	Glycans compositions
933.3	[Hex]3[HexNAc]2	1462.5	[Hex]5[HexNAc]3
1095.3	[Hex]4[HexNAc]2	1501.5	[Hex]4[HexNAc]4
1136.4	[Hex]3[HexNAc]3	1518.4	[Hex]3[HexNAc]5
1152.3^a^	[Hex]3[HexNAc]3	1664.4	[Hex]5[HexNAc]4
1257.4	[Hex]5[HexNAc]2	1705.5	[Hex]4[HexNAc]5
1298.4	[Hex]3[HexNAc]3	1745.8	[Hex]3[HexNAc]6
1339.4	[Hex]3[HexNAc]4	1866.8	[Hex]5[HexNAc]5
1355.4^a^	[Hex]3[HexNAc]4	1908.2	[Hex]4[HexNAc]6
1419.1	[Hex]6[HexNAc]2	1949.2	[Hex]3[HexNAc]7

^a^[M+K]^+^ ion peak

*Hex* Hexsose, *HexNAc* *N*-acetyl hexosamine, *Fuc* fucose

### **Enrichment and analysis of human milk oligosaccharides (HMOs) by blocked PMNPs**

**Fig. 6:**
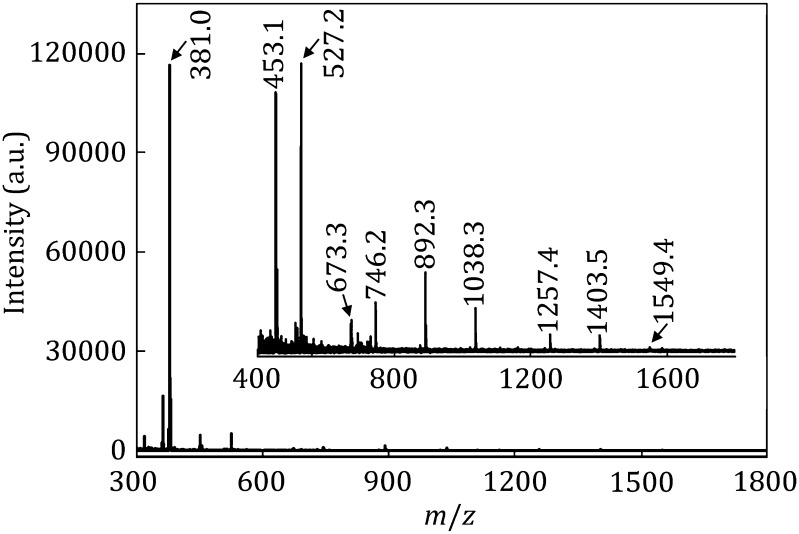
MALDI mass spectra of free oligosaccharides isolated from human milk by PMNPs. The inner spectrum was the stretched MS spectrum from *m/z* 400–1750 Da

Human milk is the primary source of nutrition for newborns, the oligosaccharides contained in the milk are very important for infants’ healthy growth. In this work, human milk oligosaccharides (HMOs) were enriched and analyzed by the blocked PMNPs. 1 μL human milk was applied for the PMNPs enrichment and analyzed by MALDI-TOF MS. The MS results are shown in Fig. 6, the mass spectra presented 10 ion peaks of oligosaccharides from human milk, the ion peak with highest intensity was *m/z* 380.97, corresponding to the [M+K]^+^ ion peak of lactose, which was the most abundant sugar in human milk. The composition of others oligosaccharides was assigned according to the MS results (Table 2). Most of the oligosaccharides had a lactose core (2[Hex]), which indicated that the HMOs (human milk oligosaccharides) were synthesized from a precursor molecule—lactose. Fucose unit was presented in the structures of most oligosaccharides, which was very important nutrient substance for infants (Zivkovic et al. 2011).

**Table 2:** Compositions of human milk oligosaccharides (HMOs) isolated by the blocked PMNPs

*m/z* [M+K]^+^	Oligosaccharides compositions	*m/z* [M+K]^+^	Oligosaccharides compositions
381.0	Lactose (2[Hex])	892.3	3[Hex][HexNAc] [Fuc]
453.1^a^	[Hex][NeuAc]	1038.3	3[Hex][HexNAc] 2[Fuc]
527.2	2[Hex][Fuc]	1257.4	4[Hex]2[HexNAc] [Fuc]
673.3	2[Hex]2[Fuc]	1403.5	4[Hex]2[HexNAc] 2[Fuc]
746.2	3[Hex][HexNAc]	1549.4	4[Hex]2[HexNAc] 3[Fuc]

^a^[M+H]^+^ ion peak

*Hex* Hexsose, *HexNAc* *N*-acetyl hexosamine, *Fuc* fucose, *NeuAc* sialic acid

We also applied the non-blocked PMNPs to isolate the HMOs. As shown in Figure S8, the non-blocked PMNPs cannot enrich HMOs efficiently. In the MS spectrum, no HMOs' characteristic peak was detected, except for lactose, since lactose is the most abundant HMOs in human milk. The other peaks were ascribed to the undesired nonspecific interferents. This result indicated that the blocked molecules are also necessary for isolating real samples. To check the detection limit of the PMNPs for isolating real samples, we reduced the human milk amount to 0.1 μL, and applied for blocked PMNPs analysis. The MS spectrum is shown in Figure S9 in which less HMOs' characteristic peaks were detected, the peaks were ascribed to Lactose (2[Hex]), [Hex][NeuAc], 2[Hex][Fuc], 3[Hex][HexNAc][Fuc], and 3[Hex][HexNAc]2[Fuc], and the other HMOs in low abundance cannot be detected efficiently. When we further reduced the human milk concentration to 0.01 μL (Figure S10), just the lactose (*m/z* 381.1) and [Hex][NeuAc] (*m/z* 453.1) characteristic peaks were detected, and the MS signal appeared in a very low intensity. When we diluted the human milk to a lower concentration (0.001 μL), the MS cannot detect any characteristic peaks. From these results, the blocked PMNPs enable to enrich the high-abundance HMOs from the human milk in very low concentration. In comparison to other separation methods, such as affinity chromatography, PMNPs method can selectively enrich the HMOs in lower concentration, and just need little amount of human milk samples. The tedious desalting and separation process also is also simplified, and the enrichment and analysis can be completed in shorter time.

## **CONCLUSION**

We have explored a facile method for selective enrichment and analysis of saccharides. Fe_3_O_4_ MNPs were fabricated by coprecipitation method, and modified with phenylboronic acid and “blocking” molecules onto the particle surface, which enabled the magnetic nanoparticles to selectively bind saccharides efficiently. The PMNPs can be easily separated from the solvent by applying assistant magnet field, therefore, the enrichment process was rapid and user-friendly. Given higher surface-to-volume ratio, the nanoparticles can carry relatively larger amount of boronic acid moieties, which enhanced the detection sensitivity. The enriched saccharides together with the PMNPs can be directly detected by MALDI-TOF MS, without further eluting steps, different from currently widely used affinity columns. This avoided unnecessary sample loss and helped to lower the detection limits of this enrichment method, for small sugar molecules detection, the detection limits were reached to pmol level. Furthermore, we successfully validated the method to identify the *N*-glycans and human milk oligosaccharides (HMOs) in low content. The established method provided a rapid and reliable approach for glycomic analysis.

## Electronic supplementary material

Supplementary material 1 (PDF 586 kb)
