# Supplementary material for: Phenylboronic acid-functionalized magnetic nanoparticles for one-step saccharides enrichment and mass spectrometry analysis
Source: Biophys Rep. 2015 Jul 14;1:61–70. doi: 10.1007/s41048-015-0002-3 (PMC4762129; doi:10.1007/s41048-015-0002-3)
Supplement: Supplementary file 1 — Supplementary material 1 (PDF 586 kb) [file 41048_2015_2_MOESM1_ESM.pdf]

SUPPORTING INFORMATION:

Phenylboronic Acid Functionalized Magnetic Nanoparticles for One-step Saccharides Enrichment and Mass Spectrometry Analysis

Xiangdong Xue[1, 2, *],Yuanyuan Zhao [1*] ,Xu Zhang[1] , Chunqiu Zhang[1], Anil Kumar[1], Xiaoning Zhang[2], Guozhang Zou[1, ✉] , Paul C. Wang[4], Jinchao Zhang[3], Xing-Jie Liang[1, ✉]

[1]CAS Key Laboratory for Biomedical Effects of Nanomaterials and Nanosafety, National Center for Nanoscience and Technology, Beijing 100190, China
[2]Laboratory of Pharmaceutics, School of Medicine, Tsinghua University, Beijing, 100084, China
[3]College of Chemistry & Environmental Science, Chemical Biology Key Laboratory of Hebei Province, Hebei University, Baoding 071002, China
[4]Fu Jen Catholic University, Taipei 24205, China

[*]These authors contributed equally.

✉Corresponding author. Xing-Jie Liang (liangxj@nanoctr.cn); Guozhang Zou (zougz@nanoctr.cn)

Supporting Information Available

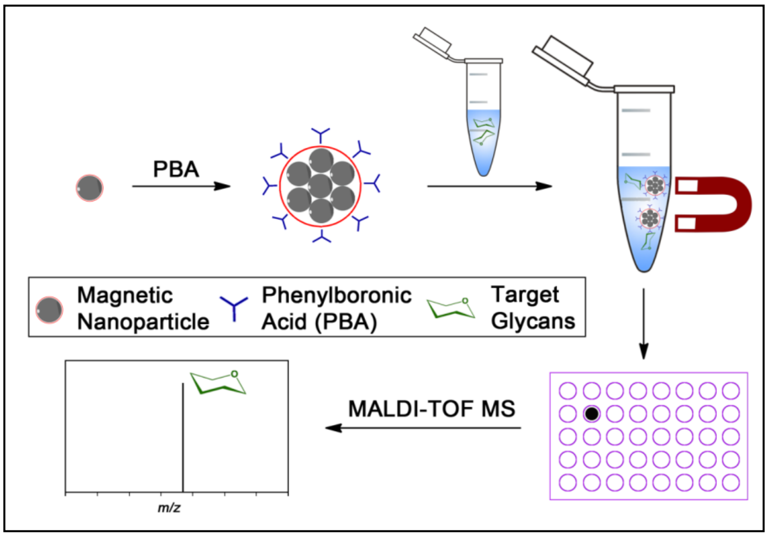

Figure S1. The One-step Enrichment of Saccharides by PMNPs and Analysis by MALDI-TOF MS.

Table S1. (Composition and structure characterization of Fe_3O_4 magnetic nanoparticles by XRD.)

Int-f	h	k	l	d(A)	Calculated d(A)
30	2	2	0	2.9670	2.9630
100	3	1	1	2.5320	2.5374
20	4	0	0	2.0993	2.1083
30	5	1	1	1.6158	1.6143
40	4	4	0	1.4845	1.4820

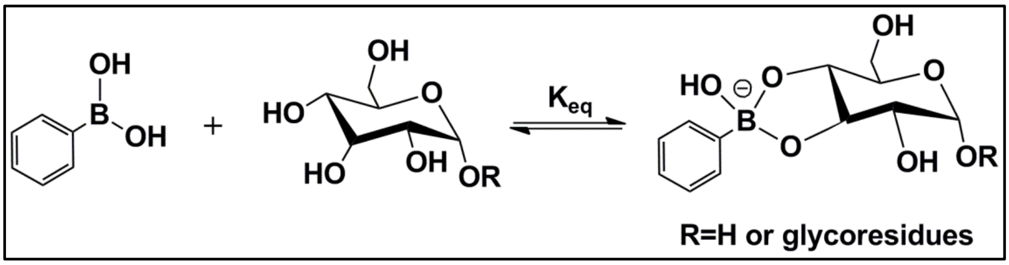

Figure S2. Boronate ester formation by reversible reaction of phenylboronic acid and glycan.

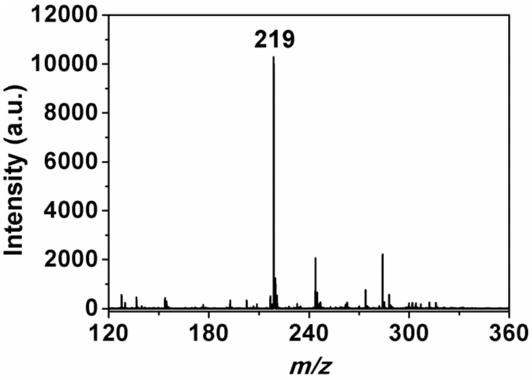

Figure S3. The MS spectrum of glucose isolated by 100 μg non-blocked PMNPs.

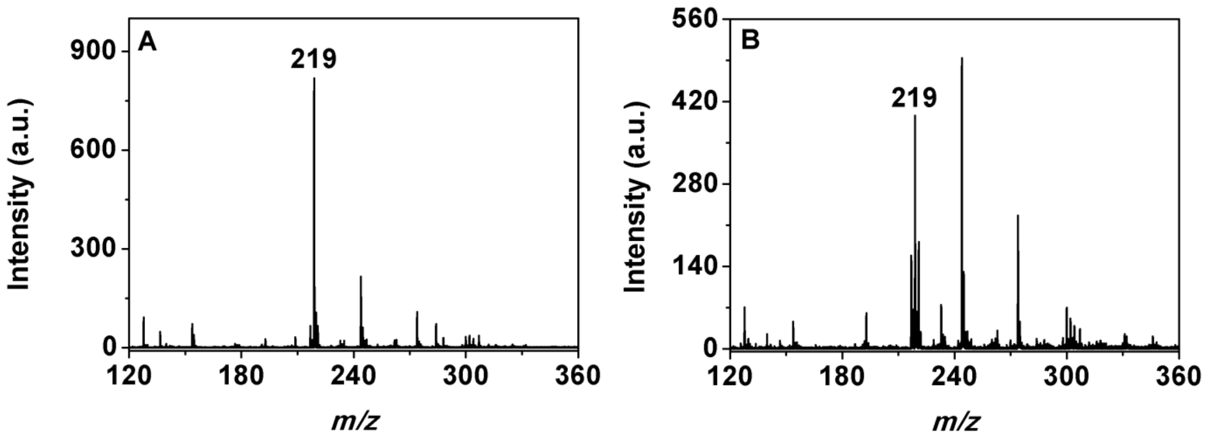

Figure S4. The MS spectra of different concentrations of glucose isolated by 100 μg blocked PMNPs. A decrement amount of glucose: A) Glucose=10^{-10} mol; B) 10^{-12} mol was mixed with glutamine in a molar ratio of 1:1. The blocked PMNPs were used to separate the glucose from glutamine, and the enriched glucose was measured by MALDI-TOF MS.

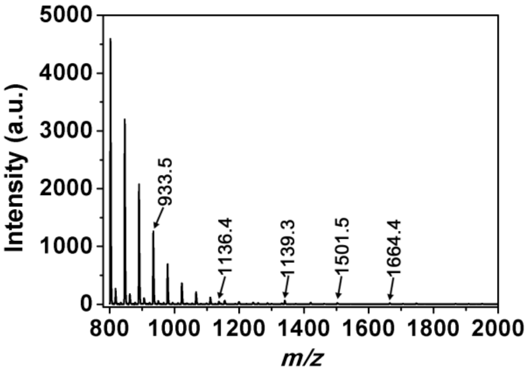

Figure S5. The MS spectrum of *N*-glycans isolated from 10 μg ovalbumin by 100 μg non-blocked PMNPs.

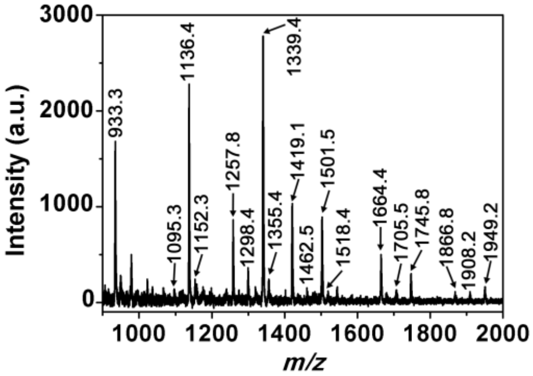

Figure S6. The MS spectrum of *N*-glycans isolated from 40 μg ovalbumin by 100 μg blocked PMNPs.

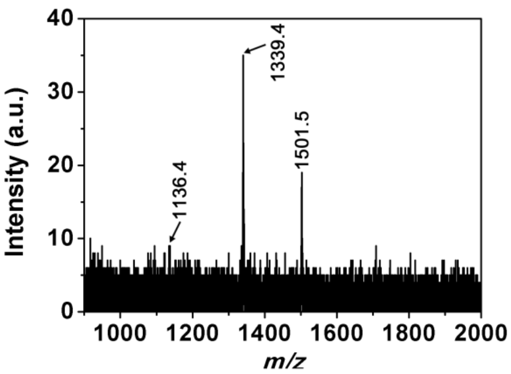

Figure S7. MS spectrum of the *N*-glycans isolated from 2 μg ovalbumin by 100 μg blocked PMNPs.

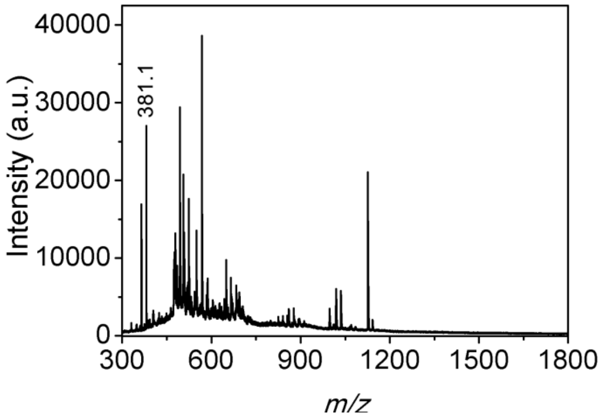

Figure S8. MS spectrum of the HMOs isolated from 1 μL human milk by 100 μg non-blocked PMNPs.

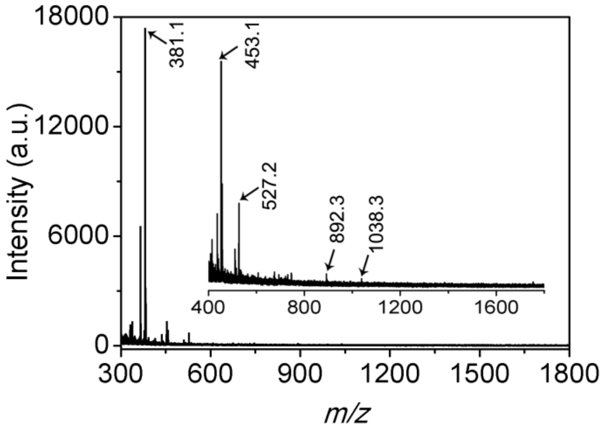

Figure S9. MS spectrum of the HMOs isolated from 0.1 μL human milk by 100 μg blocked PMNPs. The inset was the stretched MS spectrum from *m/z* 400 to 1800 Da.

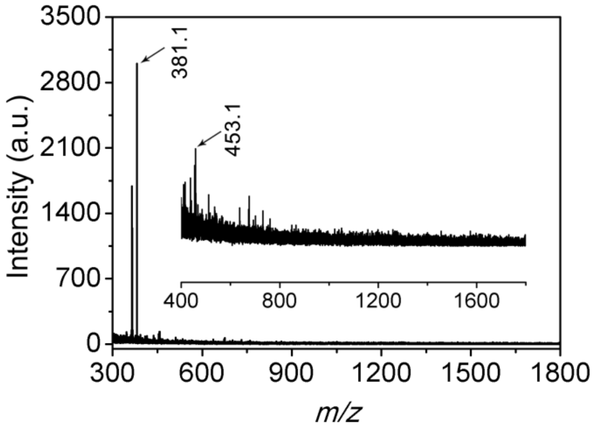

Figure S10. MS spectrum of the HMOs isolated from 0.01 μL human milk by 100 μg blocked PMNPs. The inset was the stretched MS spectrum from *m/z* 400 to 1800 Da.
